# Supplementary material for: Increasing the Hilbert space dimension using a single coupled molecular spin
Source: Nat Commun. 2021 Jul 21;12:4443. doi: 10.1038/s41467-021-24693-6 (PMC8295329; doi:10.1038/s41467-021-24693-6)
Supplement: Supplementary file 1 — Supplementary Information [file 41467_2021_24693_MOESM1_ESM.pdf]

# – Supplementary Information –

Increasing the Hilbert space dimension using a single coupled molecular spin.

Hugo Biard[a], Eufemio Moreno-Pineda[b], Mario Ruben[c,d,e], Edgar Bonet[a], Wolfgang Wernsdorfer*[a,e,f] and Franck Balestro*[a]

a. *Univ. Grenoble Alpes, CNRS, Grenoble INP, Institut Néel, 38000 Grenoble, France.*
b. *Depto. de Química-Física, Escuela de Química, Facultad de Ciencias Naturales, Exactas y Tecnología, Universidad de Panamá, Panamá, Panamá.*
c. *Institute of Nanotechnology (INT), Karlsruhe Institute of Technology (KIT), Hermann-von-Helmholtz-Plats 1, D-76344 Eggenstein-Leopoldshafen, Germany.*
d. *Centre Européen de Sciences Quantiques (CESQ) within the Institut de Science et d'Ingénierie Supramoléculaires (ISIS), 8 allée Gaspard Monge, BP 70028, 67083 Strasbourg Cedex France.*
e. *Institute for Quantum Materials and Technology (IQMT), Karlsruhe Institute of Technology (KIT), Hermann-von-Helmholtz-Platz 1, D-76344 Eggenstein-Leopoldshafen, Germany.*
f. *Physikalisches Institut, Karlsruhe Institute of Technology, D-76131 Karlsruhe, Germany.*

*Correspondence to: wolfgang.wernsdorfer@kit.edu, franck.balestro@neel.cnrs.fr

DEMONTRATION OF THE HYSTERESIS AND THE MAGNETIC ANISOTROPY

The aim of this work is to access the states of the pair of nuclear spins carried by the two Tb^{3+} ions of a single molecular magnet: Tb_2Pc_3 SMM. The magnetic signatures result in the first place from the pair of electronic spins carried by the two Tb^{3+} ions. At low temperatures, considering a ferromagnetic coupling, we are in the presence of an Ising like spin, with $|+6,+6\rangle$ and $|-6,-6\rangle$ possible orientations. Under the application of an external magnetic field, they can reverse through QTM process, with a probability given by the Landau-Zener formula (100% at our sweep rates). As we saw in Fig. 2 of the main text, there are discrete magnetic field where QTM process is possible, and those field values tell us about the state of the pair of nuclear spins.

As explained in the main text (section "Methods"), in our case, the fabrication of molecular transistors is based on the electromigration process. Due to the impossibility to control the position of the molecule, obtaining a molecular transistor requires many trials. In fact, it is necessary both that the opened interstice in between the electrodes is of the same dimension as the molecule, but also that the electric coupling is optimal. Indeed, the electronic and nuclear spins are quantum systems: if the coupling is too strong, it becomes impossible to resolve the states of the pair of nuclear spins; and conversely, if the coupling is too weak, accessing their readings is not possible.

Immediately after the electromigration, a first series of measurements is performed in order to characterize the obtained sample. We start by measuring the conductance at source–drain voltage $V_{SD} = 0$, while varying the gate voltage V_G. We hope to observe one, or more, Coulomb peaks (degeneracy points), which attest the passage from one charge state to another (Coulomb blockage area), and thus the presence of a Coulomb blockade.

If this first step was successful, we then carry out a differential conductance mapping of the (V_{SD}, V_G) space, in order to visualize the shape of the Coulomb diamonds. Their slopes and charging energy E_C provide valuable insights about the size of the quantum dot and its coupling to the source and drain electrodes. In Fig. 1b, we present the coulomb map

performed on the Tb_2Pc_3 sample used in this paper. We found a ratio C_G:C_S:C_D = 1:3.7:11.4 for the electric capacitances of the equivalent electrical circuit used to model the obtained transistor,[1] with $C_G = 2.46{\times}10^{-20}$ F.

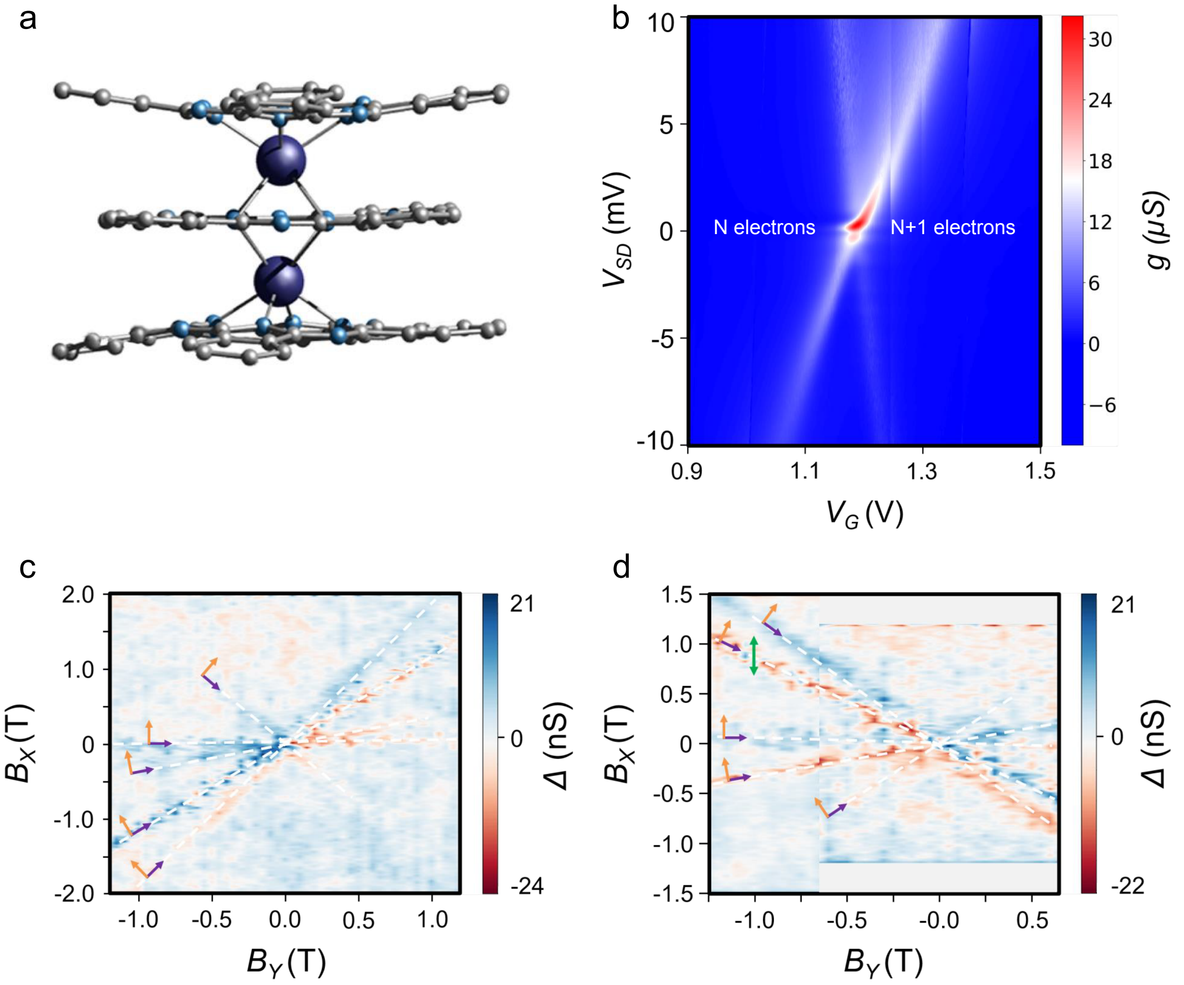

Fig. S1. (a) Side view of the Tb_2Pc_3, which can be seen as two stacked $TbPc_2$ molecules sharing a Pc plane. The magnetic properties arise from the two terbium ions Tb^{3+}, distant from each other by 3.5 Å, and surrounded by three ligands. Their electronic spins are ferromagnetically coupled to each other, allowing an indirect interaction between nuclear spins. Adapted from [2]. (b) Coulomb map of a Tb_2Pc_3 molecular transistor performed at 100 mK. The differential conductance was recorded through lock-in measurements. The shape of the diamonds plot enables us to distinguish two different charge states (N and N+1 electrons). We were also able to extract a minimum charging energy E_C = 370 meV, which is consistent with the expected value for a 1 nm-sized quantum dot.[3,4,5] (c) and (d) present a magnetic hysteresis map, for two different sample orientations relative to the magnetic coils. The gate voltage V_G is fixed and the source–drain voltage difference V_{SD} are kept at zero. For each field B_Y, the differential conductance measured on the back-and-forth sweeps are subtracted from each other, and then plotted in this map. Several coloured lines are observed, corresponding to the hysteresis zones of each anisotropic magnetic moment, evolving linearly as a function of the transverse field. We localized by orange arrows the projection of easy axes ($\mathbf{e}_\parallel$), and by purple arrows the projection of hard axes ($\mathbf{e}_\perp$), of the magnetic moments studied in the plane of the measurement ($\mathbf{B_X}$, $\mathbf{B_Y}$).

The fact that we only depict one charge degeneracy point in Fig. 1 is to prevent the device from any damages. As a result, we limit the source drain voltage V_{SD} to low values (<10mV) in order to protect the molecular magnet integrity and its stability. Indeed, at higher V_{SD}, we already observed in other devices an increase of the noise signal or even a lost of the

quantum dot signature as a higher current flowing through the molecule could irreversibly damage the transistor stability. Thus, we have not performed a Coulomb map for V_{SD} > 10mV.

In order to estimate the charging energy and the C_G, C_S, C_D. In Fig. 2, we present measurements of the differential conductance and the current as a function of the gate voltage V_G. We clearly observe two peaks corresponding to two charge degeneracy points. If we consider that the peak observed at around V_G = –6 V corresponds to the same quantum dot than the one observed around V_G = 0 V, and from the slopes of the Coulomb diamond, we can obtain an estimation of the charging energy E_C = 370 meV. Note that due to the lack of additional measurements, this is only a rough estimation performed early in the study to verify that the observed quantum dot is compatible with the dimension of the molecular system. We did not perform any studies at V_G = –5.4 V to prevent the gate dielectric from any leakage or even an irreversible damage of the molecular spin transistor.

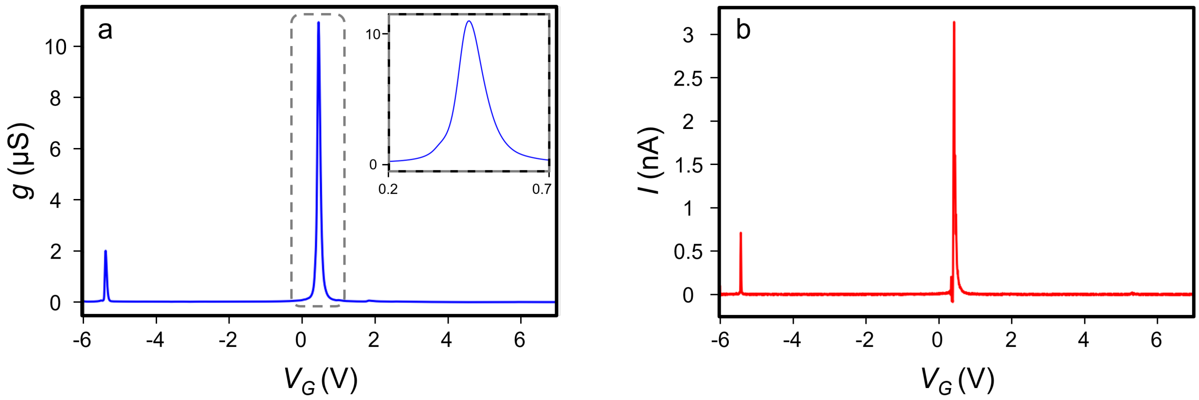

Fig. S2. Measurement of the differential conductance (a) and the current (b) as a function of the gate potential V_G.

Once the first electrical transport study is carried out, it is essential to magnetically characterize the system in order to assess that we have obtained a molecular spin transistor. The first step is to localize a gate voltage V_G working zone. In order to limit the electric current through our quantum dot, we set V_{SD} = 0. Then, close-by the degeneracy point presented in Fig. 1b, we carried out a magnetic hysteresis cartography: specifically, we recorded the differential conductance during back-and-forth magnetic sweeps along B_X, while slightly incrementing the gate voltage. The goal is to identify an area where the change in the orientation of the electronic spin pair is visible *via* an abrupt change in the conductance. Once a good V_G voltage has been found, we repeated the same measurement, but for different magnetic fields B_Y. In Fig. 1c,d, we present two magnetic maps, highlighting the hysteresis and anisotropy of the studied sample. We notice multiple hysteretic regions, corresponding to several magnetic moments. The qualitative change from one sample orientation (c) to another (d) allows us to assert that the magnetic moment also exhibits anisotropy.

At this step, we can thus assert that we have several anisotropic magnetic moments connected inside the interstice. In order to probe their possible nuclear spins states, numerous B_X back and forth magnetic sweeps were performed for a given V_G and B_Y while keeping V_{SD} = 0. The results are depicted and explained in the main text (see Fig. 3 of the main text, as well as in Fig. 3 of this supplementary information).

DIPOLAR INTERACTION

Dipolar interactions between two $J = 6$ can be calculated employing the following equation:

$$J_{dip} = \frac{\mu_B^2}{r^3} - \left[\bar{\bar{g}}_A . \bar{\bar{g}}_B - 3\left(\bar{\bar{g}}_A . \vec{R}\right).\left(\vec{R}^T . \bar{\bar{g}}_B\right)\right] \text{ (S1)}$$

where μ_B is the Bohr magneton, r is the Tb···Tb distance obtained from crystallographic analysis. $\bar{\bar{g}}_{A/B}$ is the g-matrix of ion A and B, and $\vec{R}$ is directional unit vector between the two ions. Due to the highly axial ligand field parameters, the anisotropic axis expectedly lies along the z-axis (perpendicular to the Pc planes) with no component along the x- and y-axis, therefore the g-matrix for the Tb^{3+} with $J = 6$ reads:

$$\bar{\bar{g}}_{Tb} = \bar{\bar{g}}_A = \bar{\bar{g}}_B = \begin{pmatrix} 0 & 0 & 0 \\ 0 & 0 & 0 \\ 0 & 0 & 3/2 \end{pmatrix}$$

These two Tb^{3+} ions are connected by a unit vector of the form:

$$\vec{R} = \begin{pmatrix} 0 \\ 0 \\ 1 \end{pmatrix}$$

Thus, leading to a dipolar matrix for two Tb^{3+} ions separated by a 3.5230 Å (the distance has been taken from the crystal structure in 2):

$$J_{dip} = \begin{pmatrix} 0 & 0 & 0 \\ 0 & 0 & 0 \\ 0 & 0 & +0.064 \end{pmatrix} \text{K}$$

Note that, with $J_{dip\parallel} = 0.064$ K, the ferromagnetic and anti-ferromagnetic states have a crossover at 0.4 T.[2] Experimentally, however, a coupling value of $C = 0.092$ K (i.e. $C = J_{dip} + J_{ex} = 0.092$ K where $J_{ex} = 0.028$ K) is required to reproduce the crossing at 0.55 T, which could account for some exchange occurring between the Tb^{3+} ions.[2]

ELECTRONIC STRUCTURE

For the calculation of the electronic structure of Tb_2Pc_3, we have employed the following Stevens factors and the ligand field parameters:

Table S1. Stevens factor employed for Tb_2Pc_3.

u_2	u_4	u_6
-1/99	2/16335	-1/891891

Table S2. Ligand field parameters utilised for Tb_2Pc_3.

	$A_2^0\langle r^2\rangle$	$A_4^0\langle r^4\rangle$	$A_4^4\langle r^4\rangle$	$A_6^0\langle r^6\rangle$	$A_6^4\langle r^6\rangle$
Site 1	416.37 k	-300.69 k	989.84 k	30.44 k	676.20k
Site 2	421.26 k	-283.43 k	1241.62 k	21.75 k	513.91 k

EXPERIMENTAL STATISTIC AND CORRECTION OF THE COIL'S HYSTERESIS

The next step of the study is to demonstrate the presence of the pair of nuclear spins. As explained in the main text, each of the two terbium nuclear spins is coupled to its electronic spin. The electronic spins are themselves coupled together, giving 16 different nuclear spins

states, among which 10 are not energetically degenerate. Thus, in the Zeeman diagram 10 anti-crossings allow the reversal of the electronic spins. Nevertheless, at three of these positions the electronic spins reversal occurs at the same magnetic field $B_{\parallel}$, therefore, only seven positions in magnetic field where QTM occurs should be observed.

To demonstrate the read-out of the Tb_2Pc_3 molecular magnet, we first performed a 6,000 back and forth magnetic sweeps in the sample orientation depicted in Fig. 1d of this supplementary information. We empirically found that the best signal was obtained with a high constant transverse field B_Y. The presence of other magnetic moments in the close environment, possibly arising from other molecular magnets, deteriorate the signal quality, and constrained us to apply a strong constant field along B_Y to separate their easy axes. However, the Foucault's current (or Eddy current) generated by the magnetic sweeps along B_X increase with the magnetic field B_Y, and thus deteriorate the recorded differential conductance signal. For those reasons, the statistic depicted in Fig. 3 of the main text and in Fig. 3 of this supplementary information was performed at $B_Y = -1$ T, over a range of $B_X = \pm 300$ mT, as localized by the green double arrow in Fig. 1d.

As depicted in Fig. 3a,b, of this supplementary information, for each of the 6,000 back and forth magnetic sweeps, the abrupt jump position was recorded and plotted in an histogram for the forward (a) and backward (b) sweeps. We observe for the forward sweep a higher population for the positive magnetic values, while for the backward sweep it is the case of the negative values. This is due to the relaxation of the nuclear spin system between each pass through the 7 anti-crossing positions. As one can observe in the Zeeman diagram, when the magnetic field is swept from negative to positive values, the anti-crossings associated with the lower energies correspond to the positive values, while when the magnetic field is swept from positive to negative values, the lower energies correspond to the anti-crossings at the negative values.

Knowing the peak positions in the histograms of the forward (a) and backward (b) sweeps, we were able to correct the hysteresis of the B_X coil: thus, all the histograms are centred to zero. The forward and backward sweeps are pooled together in Fig. 3c, with a recall of the Tb_2Pc_3 Zeeman diagram and its anti-crossing positions. As explained in the main text, the mismatch between the magnetic field values of the Zeeman diagram and the ones of the histograms is due to the misalignment between the easy axis of the molecule $\mathbf{e}_{\parallel}$ and the axis of the coil $\mathbf{B_X}$. Also, the combination of the forward and backward sweeps in the same histogram increases the number of events, and mostly compensates the relaxation between each pass through the 7 anti-crossings zone.

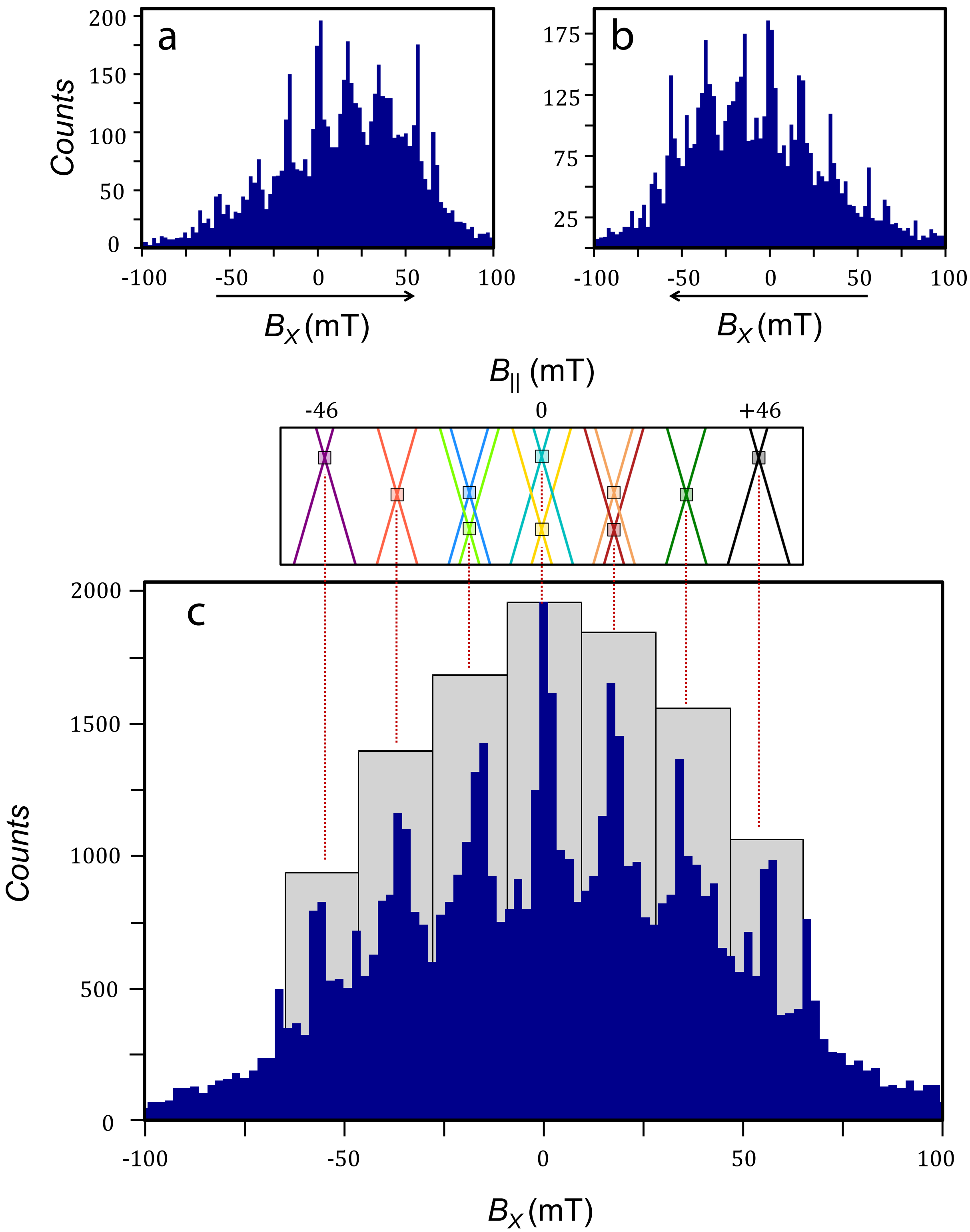

Fig. S3. The histograms (a) and (b) correspond to the differential conductance jump positions identified during magnetic back and forth sweeps, while (c) is the pooling of the two. We corrected the hysteresis of the coils and centred the histograms on zero. Above the histogram (c), the Zeeman diagram of Tb_2Pc_3 is shown for reference (within $B_{||} = \pm 55$ mT and $E/k_B = \pm 50$ mK), which helps identifying the nuclear spin transition corresponding to each peak. We can easily match the peak positions to the predicted anticrossing fields. The presented signatures of QTM were obtained on the right side of the charge degeneracy point depicted in Fig. 1b.

MEASUREMENT QUALITY

In order to study the stability of the Tb_2Pc_3 nuclear spins quantum system as a function of time, we plotted in Fig. 4 of this supplementary information, the position of the reversal of the pair of electronic spins as a function of time for the forward (a) and backward (b) sweeps, and the sum of the two (c), presented in Fig 3 a,b,c above. To ease the reading, we applied a Gaussian filter to accentuate the higher density zones. For the reason explained previously, the positive magnetic values have more events for the forward sweeps, and the negative values for the backward sweeps.

We can observe that the signal is relatively stable in time except for 3 different moments, where sudden deteriorations appear, without clear reason. It might be caused by the magnetic environment of the Tb_2Pc_3 molecule, as other molecular magnets change state, or a change in the measurement chain, or a pump or a compressor switching on. Also, it appears that the events corresponding to the two extra peaks are not as continuous as the 7 peaks expected by the theory. This tends to support the hypothesis that those extra peaks are due to an external perturbation.

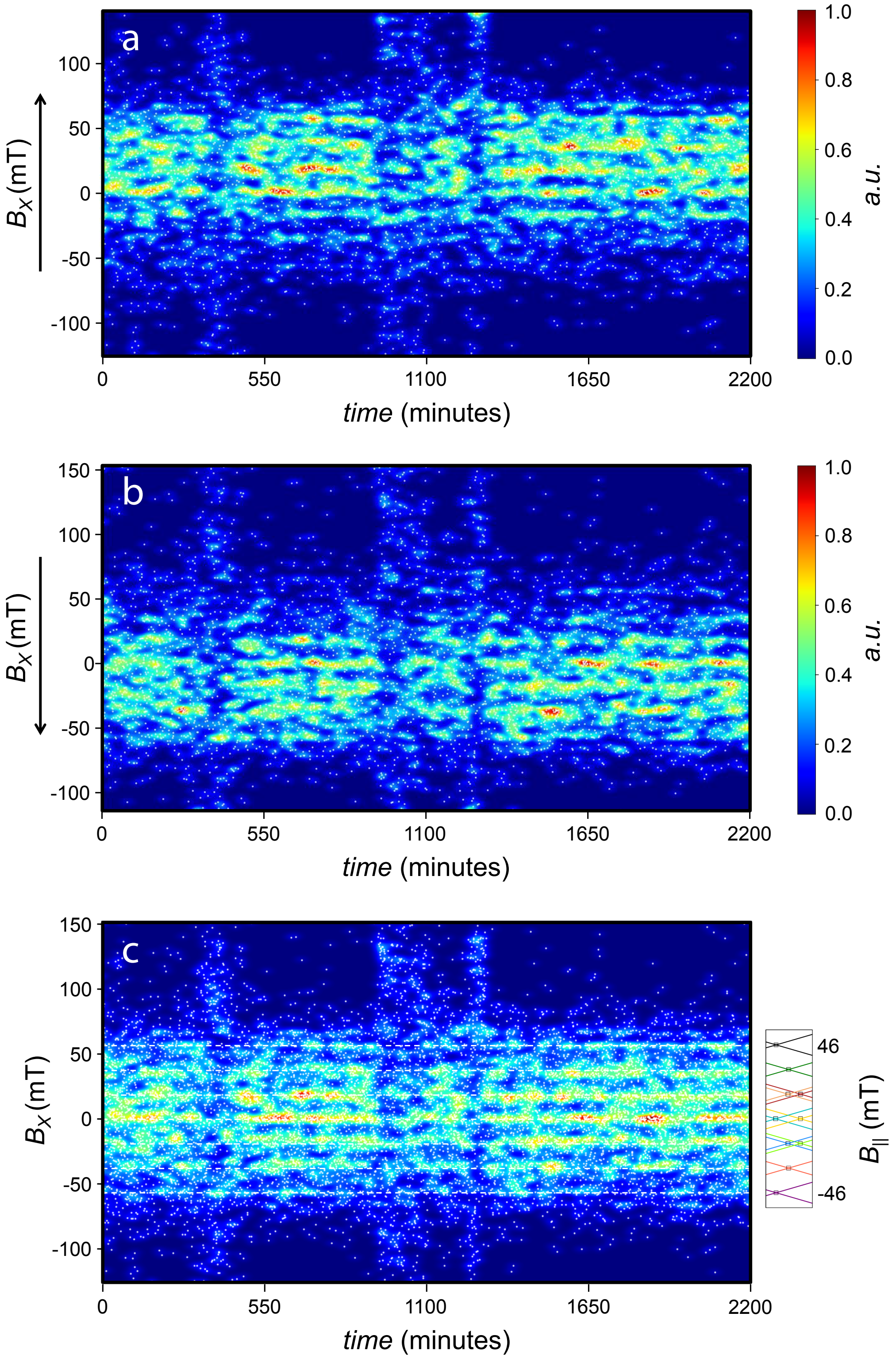

Fig. S4. Tracking of the differential conductance jump positions in time, for the forward (a) and backward (b) sweeps, and the polling of the two (c). The white dots correspond to the positions of the jumps. A Gaussian filter helps determine the local density, which we normalize from 0 to 1, and is shown as a colour scale. We placed the Tb_2Pc_3 Zeeman diagram with dotted lines to allow a better identification of the state of the pair of nuclear spins.

It has been shown in a recent work that the coupling of a molecular magnet, in that case a $TbPc_2$, with a quantum box holding a ½ electronic spin can double the number of peaks.[5,6] Also, however unlikely, two $TbPc_2$ or Tb_2Pc_3 could be aligned and their signature could be mixed in the explored magnetic zone. In both of these cases, we would not be

measuring a single Tb_2Pc_3 molecular magnet. To prove without doubt that we have read out the pair of nuclear spins of a single Tb_2Pc_3, we present in the following the same measurement for different constant magnetic fields B_Y. As previously explained, due to the magnetic environment, the available data range was narrow. We present in Fig. 5 statistics at three different constant magnetic fields $B_Y = -0.80$ T, -1.00 T (already depicted in Fig. 3c), and -1.25 T. Using the same methodology as previously, we corrected the coil hysteresis and centered the statistic on $B_X = 0$. As we can see, in all three cases, seven peaks are observed for the seven anti-crossing positions.

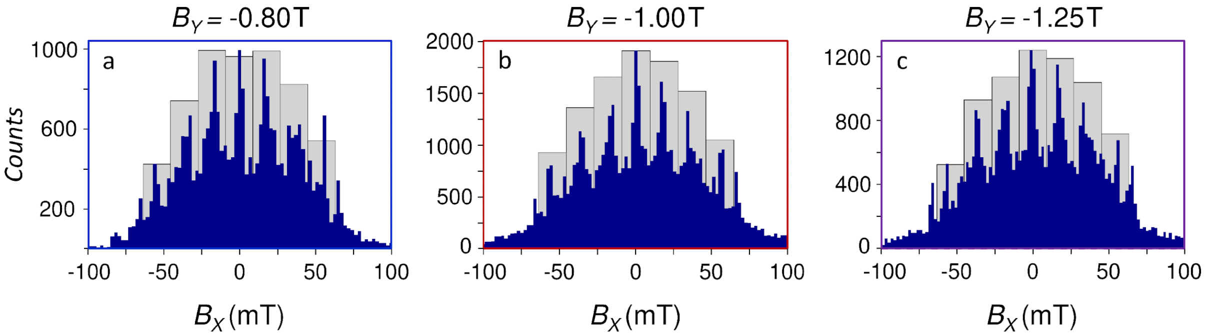

Fig. S5. Histograms of the jump positions made under three different B_Y static fields, in the orientation depicted in Fig 1d. From left to right, the transverse fields are -0.80 T for (a), -1.00 T for (b), and -1.25 T for (c); the number of back-and-forth magnetic sweeps are respectively 3,000, 6,000 and 3,800; the scanning speed is the same for all three: 50 mT/s. Although we present B_X between ±100 mT, the ranges of scans are 300 mT for (a) and (c) and 500 mT for (b). For each histogram, we can notice the good agreement with the theoretical Zeeman Diagram, which evidences the stability of the relative peak positions. Thus, we can assert that all peaks correspond to the same Tb_2Pc_3 SMM.

As for the statistic giving the histogram depicted in Fig. 3c, we plot the tracking of pair of electronic spins as a function of the time for the three different constant magnetic fields B_Y : the result, depicted in Fig. 6, shows the perfect correspondence between the three statistics, and thus confirms the presence of a single Tb_2Pc_3 molecular magnet.

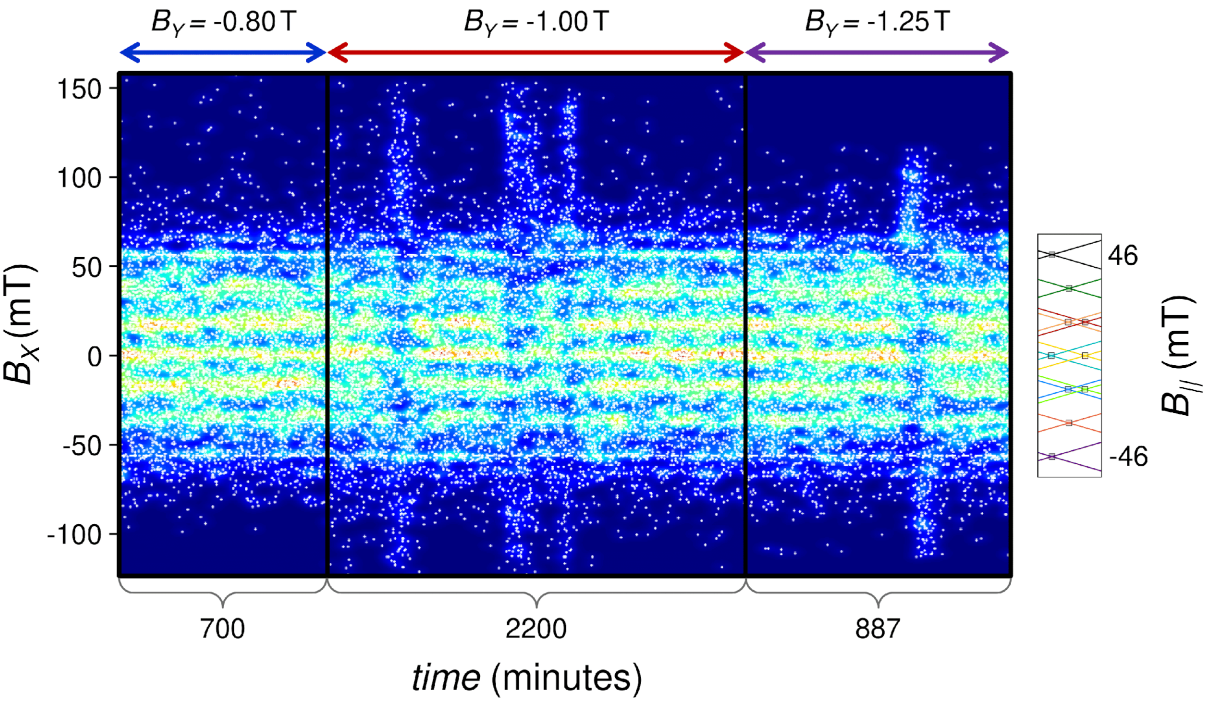

Fig. S6. Tracking of the differential conductance jump positions, as a function of time, for three different constant applied fields B_Y. They correspond to the histograms of (a), (b) and (c) depicted in Fig. 5. In order to highlight areas of higher densities, we applied a Gaussian filter. The white dots correspond to the positions of the jumps. Finally, the Tb_2Pc_3 Zeeman diagram and white dotted lines help identify the nuclear spins states. The colour bar is the same as for the tracking shown in Fig. 4.

We also propose an explanation for the wide background of events around the seven peaks. As shown in the Coupled Nuclear Spin Dynamic part of the main text, the quantum states of the pair of nuclear spins have a lifetime that is shorter than our measurement time. As explained with Fig. 7 of this supplementary information, the pair of nuclear spins can change state after passing the first anti-crossing. In that case, the quantum system can switch from a ground state (blue) to an excited state (red), opening the possibility of a direct relaxation; the latter, causing an abrupt jump in the recorded differential conductance. This could result in adding direct relaxation during the magnetic sweeps across the seven anti-crossing zone and thus the wide background of events.

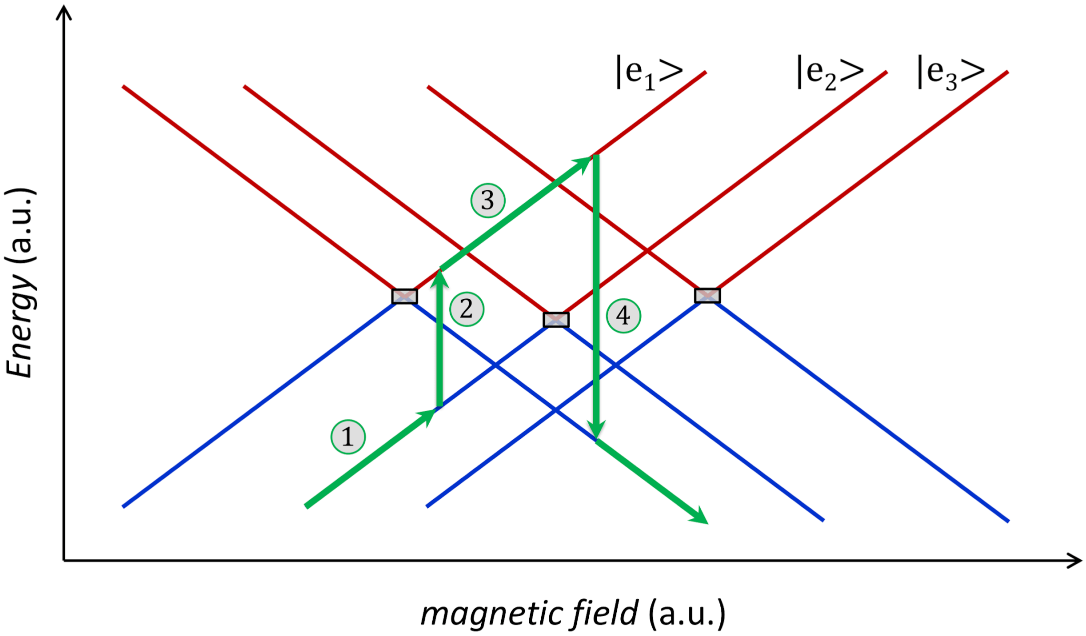

Fig. S7. The diagram presents a scenario that could possibly lead to a direct relaxation. We assume the tunnelling time in the anti-crossing zones (black rectangles) is short compared to the time required to cross those zones. We also assume the nuclear spins are initially in state $|e_2\rangle$ (1). By sweeping the magnetic field from the left to the right, the nuclear spin pair can then change its state to $|e_1\rangle$ after passing the first anti-crossing (2), thus changing from a ground state (blue lines) to an excited one (red lines). Further (3), the pair of electronic spins can then perform a direct relaxation (4) without changing the nuclear spin state. Thereby, a conductance jump is detected and an event is recorded in the histograms presented in Fig. 3 and in Fig. 5 of this Supplementary Information.

References:

1. Hanson, R., Kouwenhoven, L. P., Petta, J. R., Tarucha, S., and Vandersypen, L. M. Spins in few-electron quantum dots. Reviews of Modern Physics, vol. 79, no. 4, pp. 1217-1265 (2007).

2. Moreno-Pineda, E., Klyatskaya, S., Du, P., Damjanović, M., Taran, G., Wernsdorfer, W., and Ruben, M. Observation of Cooperative Electronic Quantum Tunneling: Increasing Accessible Nuclear States in a Molecular Qudit. Inorganic Chemistry. vol. 57, no. 16, pp. 9873-9879 (2018).
3. O'Neill, K., Osorio, E. A., and Van der Zant, H. S. J. Self-breaking in planar few-atom au constrictions for nanometer-spaced electrodes. Applied Physics Letters, vol. 90, no. 13 (2007).
4. Taychatanapat, T., Bolotin, K. I., Kuemmeth, F., and Ralph, D. C. Imaging electromigration during the formation of break junctions. Nano Letters. vol. 7, no. 3, pp. 652-656 (2007).
5. Roch, N. Transistors à molécule unique : des effets Kondo exotiques à la spintronique moléculaire, PhD thesis, Université Joseph Fourier, 2009.
6. Vincent, R. Spintronique moléculaire : étude de la dynamique d'un spin moléculaire unique. PhD thesis. Université de Grenoble (2012).
7. Urdampilleta, M., Klyatskaya, S., Ruben, M. and Wernsdorfer, W. Magnetic Interaction Between a Radical Spin and a Single-Molecule Magnet in a Molecular Spin-Valve. ACS Nano. 9, 4, 4458–4464 (2015).
